# Lower Limb Lymphoedema as an Unusual Initial Presentation of Metastatic Prostate Cancer

**DOI:** 10.7759/cureus.104978

**Published:** 2026-03-10

**Authors:** Deen M Khalil, Waleed Amjad, Anurup Kumar, Mohamed Malik

**Affiliations:** 1 Oncology and Haematology, Hull University Teaching Hospitals NHS Trust, Hull, GBR; 2 Internal Medicine, Hull University Teaching Hospitals NHS Trust, Hull, GBR; 3 Diabetes and Endocrinology, Northern Lincolnshire and Goole NHS Foundation Trust, Scunthorpe, GBR

**Keywords:** diagnostic imaging, lymphatic diseases, lymphedema, lymph nodes, neoplasm metastasis, prostatic neoplasms

## Abstract

Secondary lymphoedema in malignancy typically arises as a result of its treatment. In rare cases, it can be a manifestation related directly to the metastatic disease, through lymphatic obstruction or infiltration. Lymphoedema as the initial presentation of advanced prostate cancer has been described only in isolated case reports.

We present an unusual case of a 66-year-old obese male with type 2 diabetes mellitus and hypertension, who presented with a seven-year history of progressive bilateral lower limb swelling, repeatedly diagnosed as cellulitis secondary to idiopathic lymphoedema. He had received multiple courses of antibiotics for recurrent Group G Streptococcal cellulitis. On admission, he was febrile with malodorous, oedematous lower limbs displaying papillomatosis and fibrotic, cobblestone-like plaques. Imaging revealed widespread lymphadenopathy, destructive vertebral lesions, and features concerning for metastatic malignancy. A markedly elevated prostate-specific antigen (130 μg/L) alongside MRI findings of a prostate imaging reporting and data system, category 5 (PI-RADS 5) lesion in the prostate, pelvic nodal disease, and lytic bony metastases suggested a radiological diagnosis of metastatic prostate cancer. The patient was commenced on androgen deprivation therapy following multidisciplinary team discussion and unsuitability for prostate biopsy.

To the best of our knowledge, only one case of unilateral lower limb lymphoedema as the initial presentation of metastatic prostate cancer has been reported. This case demonstrates a plausible association between bilateral lower limb lymphoedema and metastatic prostate cancer. We discuss mechanisms of lymphoedema development in malignancy, emphasise the importance of considering underlying malignancy in unexplained or refractory lymphoedema and express the need for comprehensive assessments in unexplained secondary lymphoedema to help mitigate the impact of cognitive biases.

## Introduction

Secondary lymphoedema is a chronic, often incurable disease with variable causes. In cases of cancer, it is most commonly the result of damage to a normally functioning lymphatic system following neoadjuvant treatment such as surgical excision or radiation therapy, and in some cases, adjuvant treatment [1]. However, in rare cases, secondary lymphoedema can also be the result of direct lymphatic obstruction or infiltration caused by metastatic disease [2-4]. This phenomenon has been scarcely documented in cases of lung cancer [4], breast cancer [2], leiomyosarcoma [5] and multiple myeloma [6].

Prostate cancer is one of the most common malignancies in men across the world and is the leading cause of cancer-associated mortality in the West [7]. Typically, it is a slow-growing cancer, and patients may be asymptomatic in earlier stages. Later symptoms may include urinary symptoms or complications from metastatic disease, such as back and bone pain. Inguinal lumps [8] have been demonstrated as an initial presentation of prostate cancer, and lymphoedema in the upper extremity [9] has been demonstrated as a sign of relapse in prostate cancer. However, to the best of our knowledge, there are no case series quantifying the frequency of lymphoedema as the first presentation in untreated prostate cancer and unilateral lower limb lymphoedema [10] as an initial presentation of metastatic prostate cancer has only been described in an isolated case report.

Although secondary lymphoedema is most commonly associated with oncological treatment, in current literature, its presentation as an initial manifestation of untreated malignancy is infrequently reported. Consequently, tumour-related lymphatic compromise may be under-recognised. Further documentation of such cases, particularly in older individuals with no prior cancer history, is needed to raise clinical awareness and to understand the underlying pathophysiological mechanisms. This report describes a case of chronic, bilateral, lower limb lymphoedema as the principal presenting feature of newly diagnosed metastatic prostate cancer and aims to contribute to the limited evidence base on secondary lymphoedema associated with malignancy.

## Case presentation

We present the case of a 66-year-old gentleman, Mr. A, with a background of well-controlled type 2 diabetes mellitus, hypertension and obesity, with a BMI of 32.9 kg/m². He first presented to his general practitioner seven years prior to our encounter, with gradually worsening lower limb swelling and erythema. He had no notable family history of lymphoedema and denied any foreign travel. His case was repeatedly diagnosed as cellulitis secondary to idiopathic chronic lower limb primary lymphoedema. He was treated numerous times over the prior seven years with thirteen courses of antibiotics in the last year alone, including two prior hospital attendances for a recurrent Group G Streptococcus cellulitis of the left lower limb.

**Figure 1:**
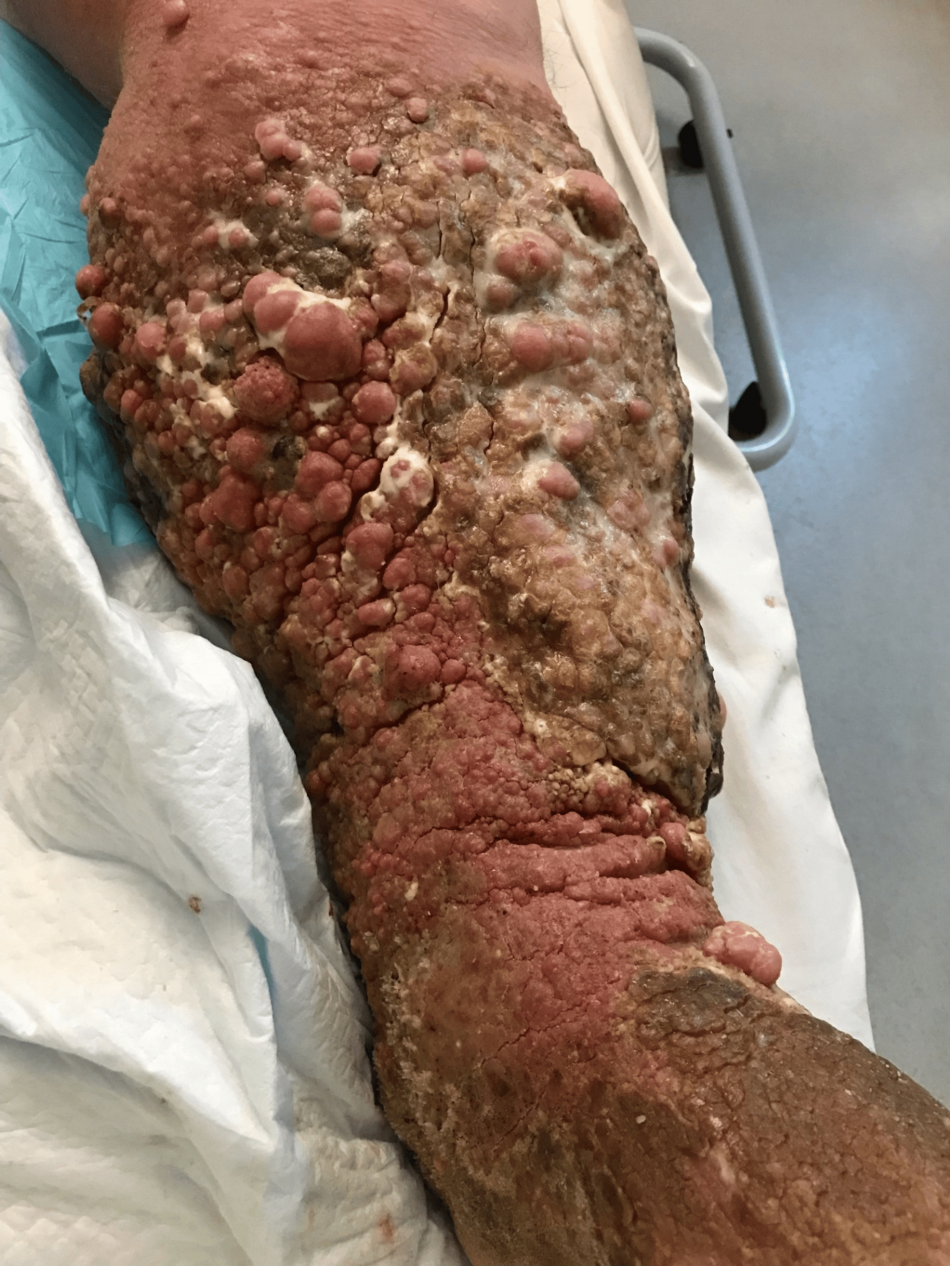
Extensive lower limb dermatological changes.

On this encounter, he presented to the emergency department in June of 2025 with complaints of increasingly painful and oedematous lower limbs, emanating a malodorous discharge. On examination of the lower limbs, extensive dermatological changes could be seen (Figure 1). The skin surfaces of the lower legs were erythematous and markedly distorted bilaterally by papillomatosis. Several exophytic and fibromatous lesions, many of which were coalescing to form a thickened, cobblestone-like plaque, extended from the proximal leg, below both knees, to the dorsum of the feet. The erythema formed a clearly demarcated line at the left mid-thigh. His abdomen was distended and hard, without guarding or rigidity; bowel sounds were audible, and there was no palpable organomegaly or lymphadenopathy. Initial biochemistry panel was unremarkable, but his white cell count was raised at 17.5 x 10^9^/L, and his neutrophil count was also raised at 16.47 x 10^9^/L, with a C-reactive protein level of 186mg/L (Table 1). Doppler ultrasound of the lower limbs was performed, excluding a deep vein thrombosis, although the examination was limited due to the extensive skin changes. He was empirically treated with antibiotics for a left lower limb cellulitis whilst awaiting microscopy, cultures and sensitivity.

**Table 1:** Initial haematology and biochemistry profiles demonstrating elevated inflammatory markers.

Investigation	Result		Reference
Haemoglobin	123 g/L	↓	130 – 180 g/L
White Cell Count	17.5 x 10^9^/L	↑	3.6 – 11.0 x 10^9^/L
Neutrophils	16.47 x 10^9^/L	↑	1.8 – 7.5 x 10^9^/L
Platelets	297 x 10^9^/L		140 – 400 x 10^9^/L
C–Reactive Protein	186 mg/L	↑	<5 mg/L
Adjusted Calcium	2.39 mmol/L		2.2 – 2.6 mmol/L
Urea	6.2 mmol/L		2.5 – 7.8 mmol/L
Creatinine	81 μmol/L		59 – 104 μmol/L
Bilirubin	24 μmol/L	↑	<21 μmol/L
Alkaline Phosphatase (ALP)	53 U/L		30 – 130 U/L
Alanine Aminotransferase (ALT)	20 U/L		<41 U/L
Albumin	26 g/L		35 –5 0 g/L

**Figure 2:**
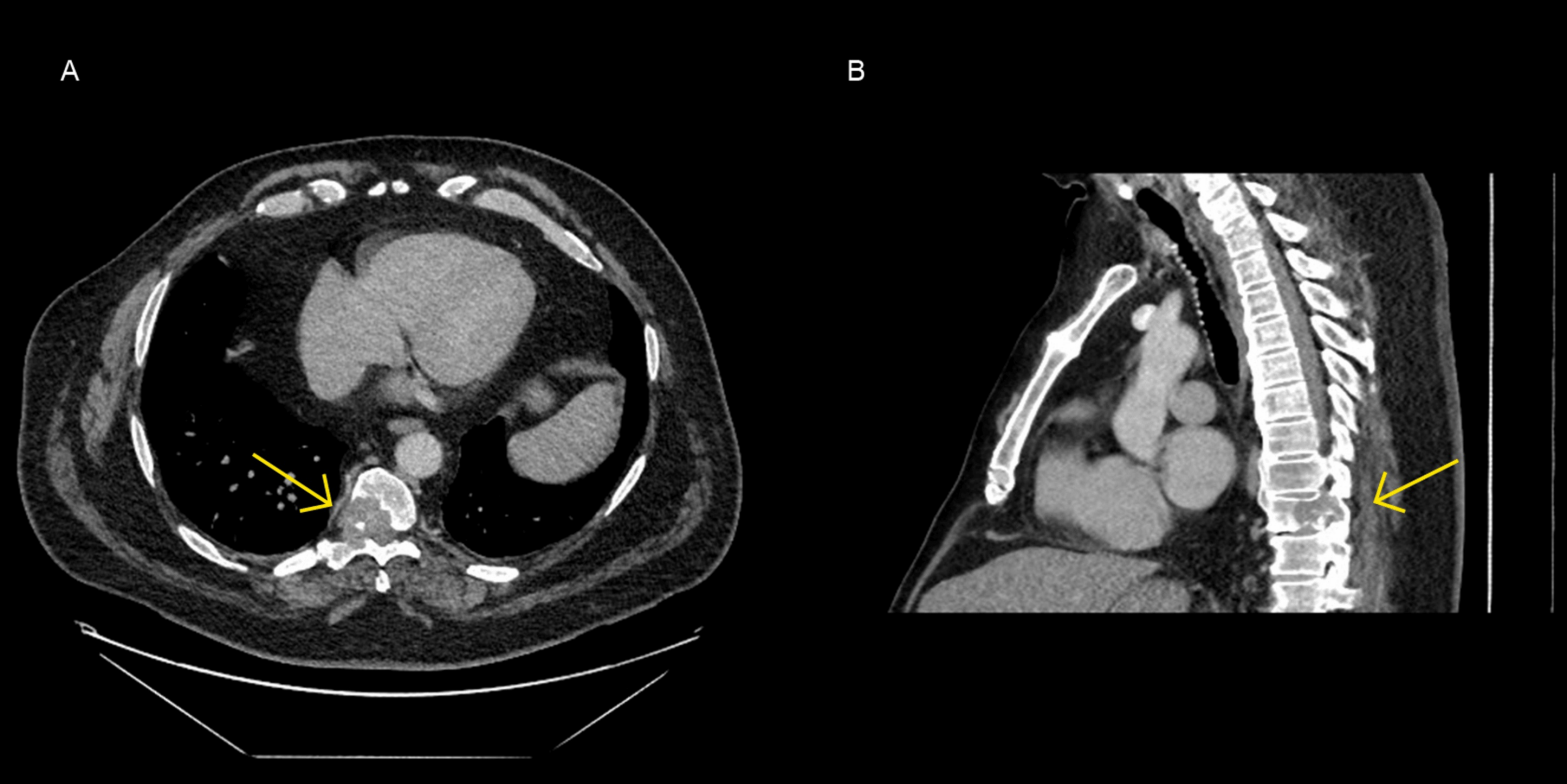
Contrast-enhanced CT of the thorax. (A) Axial and (B) Sagittal CT slices demonstrating a T9 destructive bone lesion with soft tissue involvement causing spinal stenosis (yellow arrows).

Nonetheless, an important question remained: what was the cause of the chronic lymphoedema, and could the abdominal findings point towards an intra-abdominal cause? Mr. A also explained that he had been experiencing discomfort in his lower abdomen and back. He denied any recent unintentional weight loss, fevers or night sweats. Considering this, a contrast-enhanced CT Thorax, Abdomen and Pelvis was performed (Figure 2). This identified enlarged right hilar, retroperitoneal, bilateral external iliac and left inguinal lymph nodes. A destructive bone lesion was noted in the T9 vertebral body extending into the right pedicle, with a soft tissue component causing narrowing of the spinal canal, and a metastatic lesion was noted in the T4 vertebral body. No primary tumour could be identified within the thorax, abdomen or pelvis.

**Figure 3:**
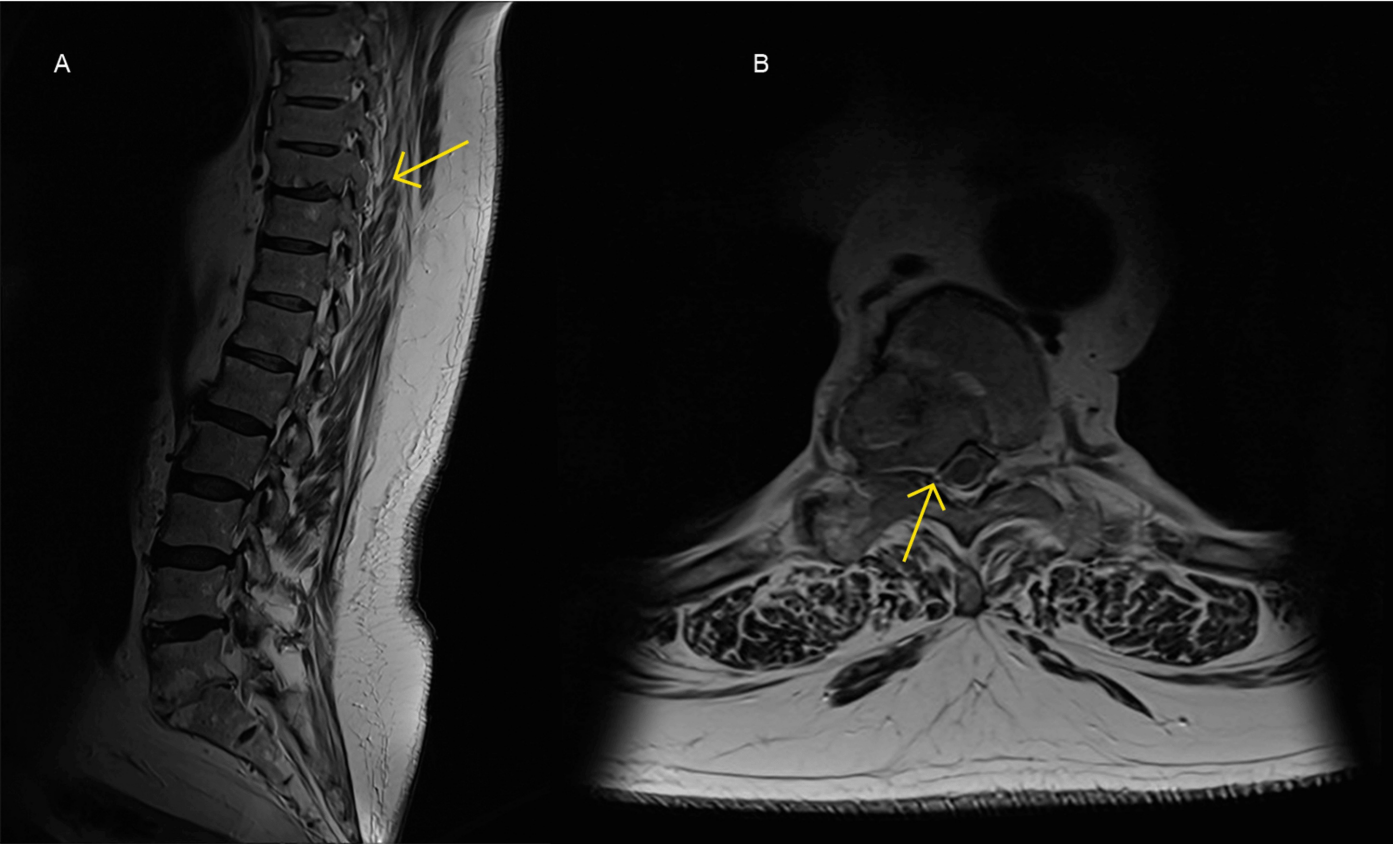
T2-weighted MRI of the thoracolumbar spine. (A) Sagittal T2 slice demonstrating multiple spinal lytic lesions with destruction of the T9 vertebra (yellow arrow); (B) Axial T2 slice at the level of T9, demonstrating expansion of the T9 vertebral body resulting in flattening of thecal sac (yellow arrow).

Considering the radiological findings and the suspicion for impending spinal cord compression, a neurological examination was performed. Although the lower limbs could not be adequately assessed given the skin changes and lymphoedema, there were no focal neurology deficits, with power, reflexes and sensation intact bilaterally. To further characterise the CT findings, an MRI Whole Spine and Brain were performed (Figure 3) and demonstrated multiple lytic lesions at C2, C7, T1, T4, T5, T8, T9, T12, L1 and L4, all varying in size, signal and severity, with expansion of the T9 vertebral body, pedicle and lamina, slightly flattening the thecal sac. There were a few hyperintense bony foci in the skull. However, no metastatic spinal cord compression or compressive myelopathy was visualised. Again, no discernible primary tumour could be seen. In light of the findings, our differential diagnoses extended to a probable prostate cancer, with multiple myeloma a feasible alternative, although he only had a mild anaemia with a haemoglobin of 123 g/L, normal renal function tests, normal serum calcium and alkaline phosphatase levels. This suggested a mixed or evolving metastatic pattern rather than purely osteolytic disease.

A prostate-specific antigen (PSA) level was tested, and a myeloma screen was sent for analysis. The myeloma screen returned with an elevated serum kappa (52.6mg/L) and lambda (46.9mg/L), with a normal kappa: lambda ratio (1.12) (Table 2). Protein electrophoresis did not identify any monoclonal paraprotein bands. These results were consistent with an acute phase response, and thus, multiple myeloma became a less likely differential diagnosis.

**Table 2:** Initial myeloma screening consistent with an acute inflammatory response.

Investigation	Result		Reference
Serum Kappa	52.6 mg/L	↑	3.3 – 19.4 mg/L
Serum Lambda	46.9 mg/L	↑	5.7 – 26.3 mg/L
Kappa : Lambda Ratio	1.12		0.26 – 1.65
IgG	19.62 g/L	↑	6.0 – 16.0 g/L
IgA	5.15 g/L	↑	0.8 – 3.0g /L
IgM	0.29 g/L	↓	0.4 – 2.5 g/L
Beta–2–Microglobulin	2.6 g/L	↑	0.7 – 1.8 mg/L
U. Bence Jones Proteins	Negative		
Protein Electrophoresis	No monoclonal paraprotein bands seen		

A digital rectal examination was performed for prostate assessment, and a smooth palpable prostate was identified. Upon further clarification, Mr. A revealed that he had been recently experiencing urinary hesitancy, post-micturition dribble and nocturia. More significantly, PSA sent prior to examination returned as 130 μg/L. At this stage, Mr. A’s inflammatory markers had improved greatly with empirical antibiotic therapy. Cultures and sensitivity from the cellulitic legs showed a growth of Group G Streptococci, sensitive to penicillin. He was subsequently discharged with an additional course of antibiotics, a two-week wait outpatient multiparametric MRI Prostate, and arranged to be discussed in the urology multidisciplinary team (MDT) meeting.

**Figure 4:**
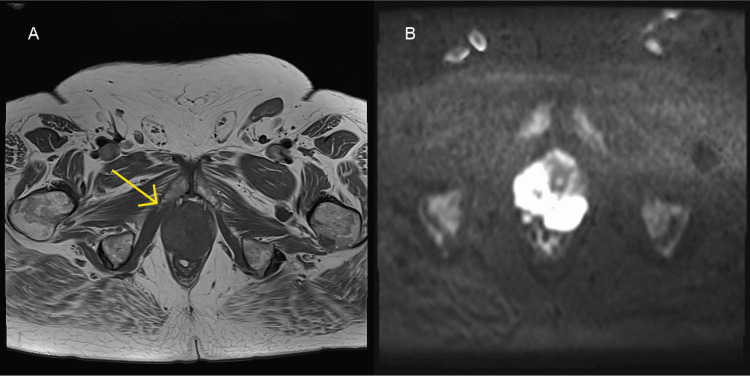
Multiparametric MRI of the prostate. (A) Axial T2-weighted slice demonstrating a focal hypointense lesion at the apex of the prostate (yellow arrow); (B) Diffusion-weighted image demonstrating hyperintensity at the same location consistent with restricted diffusion.

**Figure 5:**
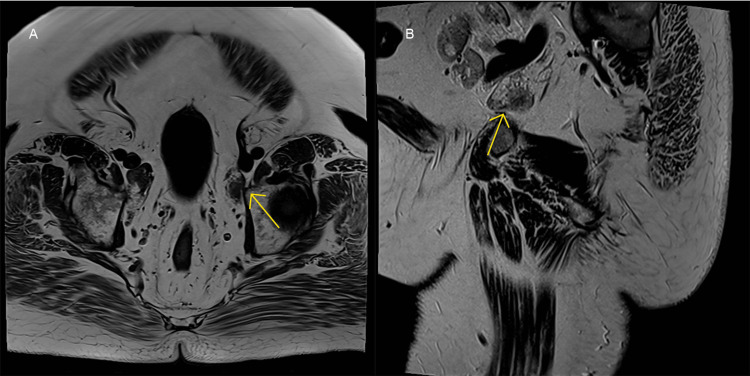
T1-weighted MRI of the pelvic lymph nodes. (A) Axial T1-weighted and (B) coronal T1-weighted slices demonstrating an enlarged, 3.6cm left sided lymph node within the external iliac region (yellow arrows).

Multiparametric MRI Prostate (Figure 4) identified a 6.2 x 4.6 x 5.2 cm prostate, with a PSA density of 1.6 (prostate volume 77ml). A lobulated, T2 hypointense mass lesion with diffusion restriction was seen from the 4 o’clock to 12 o'clock position at the apex of the prostate, corresponding to PI-RADS 5 [11]. Capsular breach was suspected, without definitive features of invasion. There were multiple enlarged bilateral external iliac and common iliac lymph nodes anatomically contiguous with the external iliac vessels, measuring up to 3.6 cm on the left side (Figure 5).

**Figure 6:**
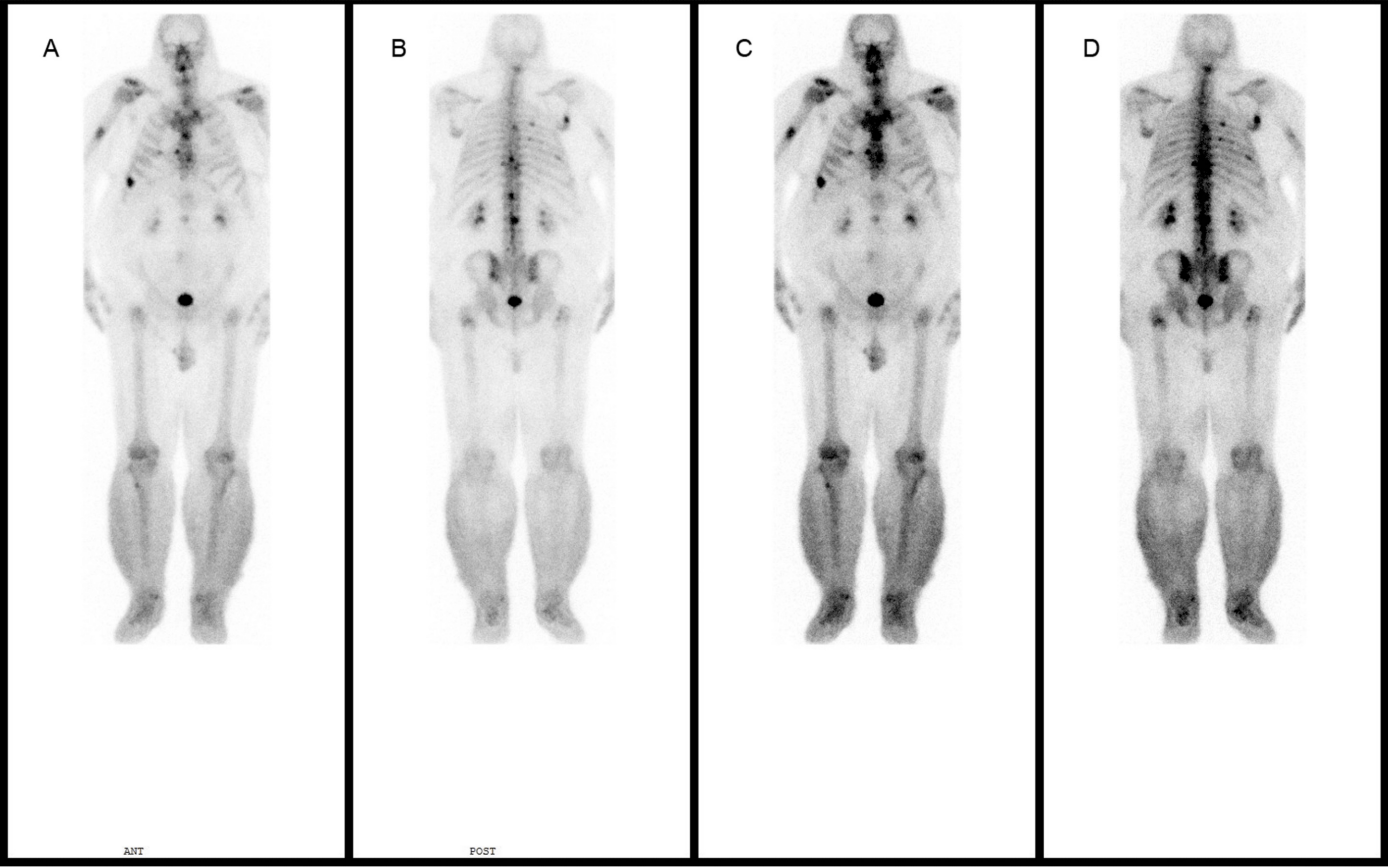
Whole-body Tc99m bone scintigraphy. (A) Anterior and (B) posterior projections demonstrating increased uptake in multiple ribs, spine and right humerus. (C) Anterior and (D) posterior projections demonstrating further areas of increased uptake to the spine, pelvis and proximal femora.

A Tc99m whole body bone scan was performed, and identified several foci suspicious for skeletal metastases in multiple bones, including the right humerus, right scapula, several ribs anteriorly and posteriorly, thoracic and lumbar spine, and the pelvis and proximal femora (Figure 6). Functional lymphatic imaging was not available within our institution, and therefore, direct visualisation of lymphatic obstruction could not be achieved.

Following MDT discussion, a CT-guided biopsy was arranged of the left external iliac lymph node. As the lymph node had reduced in size, with fatty hilum preservation, the biopsy was not completed. Due to his body habitus, he could not tolerate a prostate biopsy transperineally or transrectally.

It was subsequently decided at MDT that due to the advanced stage of the cancer, he would commence a gonadotropin-releasing hormone (GnRH) receptor antagonist without a tissue diagnosis. He commenced neoadjuvant therapy with relugolix in August 2025, eventually opting to discontinue treatment in November 2025 due to undesirable side effects and the emotional burden of the disease. He was discharged from urology care in January 2026 without further treatment or investigation.

## Discussion

Overview

Lymphoedema describes the accumulation of protein-rich lymph fluid within the extracellular space, commonly affecting the extremities [12]. Patients may present at different stages of disease, ranging from soft, pitting swelling in early spontaneous stages, to fibrotic skin changes prone to infection and non-pitting swelling in lymphostatic elephantiasis. In the earlier stages, swelling is often reversible and responds well to compression, whereas later stages become irreversible and non-responsive to conservative management.

Cases of lymphoedema can be subdivided into primary and secondary disease. Primary disease is a rare occurrence affecting 1 in 100,000 individuals, presenting most commonly within 2 years of birth, and is the result of abnormal lymphatic development [13]. Secondary disease often results from damage or obstruction to a normally functioning lymphatic system [1]. Cases are normally due to malignancy or its treatments, such as surgical excision or radiation therapy.

Cognitive bias

In the case of Mr. A, given his presentation with a gradually developing lymphoedema in the sixth decade of life, and with no significant family history or past medical history, a primary lymphoedema was seemingly less likely. Hence, it was clear that further consideration of a secondary cause for the lymphoedema was needed. Although Mr. A. had no typical “B symptoms”, it was not unreasonable to consider an underlying malignancy as a cause, given his complaints of back and abdominal pain.

The impact of cognitive bias in diagnostic decision-making has been well documented, and with relevance to Mr. A.’s case, the impact of availability bias and diagnostic momentum is especially important to note. Availability bias describes occurrences where a diagnosis appears more likely if it comes to mind faster, and this influences diagnostic momentum, where if a diagnosis is readily available, it becomes a label attached to the patient [14]. In context, Mr. A.’s lymphoedema was diagnosed as idiopathic very early on, and this became the ‘label’ at subsequent consultations. However, once Mr. A. began to develop cellulitis, for which he was a frequent attender to the primary care physicians, the label soon changed to recurrent cellulitis, but the root cause for the recurrence was not revisited until now. Considering this, his lower urinary tract symptoms were not explored as they may have appeared seemingly irrelevant to his presenting complaint. However, diagnostic momentum is multifactorial. Most importantly, time constraint pressures such as primary care consultation windows and acute medical post-take volume can impact this heavily [15].

Lymphoedema in malignancy

Malignancy typically results in lymphoedema because of its treatment, including radical dissection, lymph node clearance and radiotherapy [16]. Most notably, this is well documented in breast cancer cases, and its identification and management have been extensively researched [17]. However, the incidence of lymphoedema resulting directly from a malignancy is less well explored. In such cases, the resulting lymphoedema has been documented as being secondary to one of two pathological pathways, lymphatic infiltration (lymphangitis carcinomatosis) and lymphatic obstruction.

Cutaneous lymphangitis carcinomatosis was described by Damstra, Jagtman & Steijlen [2] as a metastatic manifestation in patients with a previous history of breast, ovarian and skin cancers. Presentations including erythematous maculae with associated lymphoedema, in both lower and upper extremities, were depicted. Histopathological examination of skin biopsies revealed clusters of tumour cells infiltrating the walls of lymph vessels. In all three cases, the presentations did not raise an early suspicion for metastatic disease, but given their malignancy backgrounds, a secondary effect of cancer treatment was given first consideration. This mechanism of lymphatic infiltration resulting from prostate cancer was demonstrated by Tang, Campbell & Pattison [3], who detected diffuse lung parenchymal Prostate-Specific Membrane Antigen uptake representing lymphangitic carcinomatosis.

Lymphatic obstruction refers to the direct, mechanical blockade of the lymphatic flow by a malignancy, either externally or by infiltration of lymph nodes, which results in the obstruction of lymph flow. A 2016 review of two cases of lung adenocarcinoma described lymphatic obstruction as a result of intra-abdominal lymph node, pelvic bone and femoral metastases resulting in lower limb lymphoedema [4]. However, as patients had undergone treatment, it was difficult to ascertain whether the lymphoedema was solely due to the metastatic disease as opposed to having influence from the chemotherapy. Adhikari, Sharan & Khumukcham [10] also demonstrated a pattern of bilateral inguinal nodal metastases, probably leading to unilateral lymphoedema in a patient histologically confirmed as being of prostatic origin; however, the exact mechanism of developing lymphoedema could not be ascertained in the absence of lymphatic imaging.

Summary and limitations

The imaging findings in Mr. A's case have suggested extensive prostatic disease with capsular breach, including involvement of the pelvic lymph nodes in anatomical proximity to the major lymphatic drainage pathways. Our findings describe those similar to Adhikari, Sharan & Khumukcham [10]. Lymphatic obstruction secondary to metastatic prostate cancer, therefore, represents the most plausible explanation for the secondary lymphoedema. Although lymphatic infiltration cannot be entirely excluded due to the absence of skin biopsies and 18F-FDG-PET-CT imaging.

We recognise that the conclusions drawn from our case are largely inferential in the absence of a histopathological diagnosis and dedicated lymphatic imaging, such as MR lymphangiography (MRL) and lymphoscintigraphy, which are highly regarded in the investigation of secondary lymphoedema in oncological patients [18].

Therefore, the association between prostate cancer and lymphoedema remains a presumption, and it is difficult to ascertain whether the early investigation of bilateral lymphoedema is correlated with an earlier detection of prostate cancer.

Further case documentation with extended histopathological evidence and lymphatic imaging is needed to better characterise the frequency, mechanisms, and clinical course of lymphoedema arising in the context of untreated prostate cancer.

## Conclusions

This case illustrates how nodal metastases from prostate cancer may initially present with chronic, bilateral, lower limb lymphoedema. Although histopathological confirmation and dedicated lymphatic imaging could not be obtained, the anatomical distribution and burden of nodal disease support malignant lymphatic obstruction as the most plausible mechanism. In older patients without a history of primary lymphatic disease who present with unexplained lower limb swelling, malignancy should be included in the differential diagnosis, and early cross-sectional imaging should be considered.

The case also highlights the potential influence of cognitive biases in prolonging diagnostic pathways. Anchoring to an earlier diagnosis of idiopathic lymphoedema contributed to delayed consideration of secondary causes. Clinicians should remain attentive to refractory or progressive presentations, as periodic diagnostic re-evaluation may reduce the risk of a missed or delayed diagnosis.
